# miRNAs contributing to the repair of tendon injury

**DOI:** 10.1007/s00441-023-03780-8

**Published:** 2023-05-30

**Authors:** Kexin Lyu, Xinyue Liu, Tianzhu Liu, Jingwei Lu, Li Jiang, Yixuan Chen, Longhai Long, Xiaoqiang Wang, Houyin Shi, Fan Wang, Sen Li

**Affiliations:** 1 School of Physical Education, Southwest Medical University, Luzhou, China; 2 Neurology Department, The Affiliated Traditional Chinese Medicine Hospital of Southwest Medical University, Luzhou, China; 3 Spinal Surgery Department, The Affiliated Traditional Chinese Medicine Hospital of Southwest Medical University, Luzhou, China; 4 Traumatology and Orthopedics Department, The Affiliated Traditional Chinese Medicine Hospital of Southwest Medical University, Luzhou, China; 5 The Affiliated Traditional Chinese Medicine Hospital of Southwest Medical University, Luzhou, China

**Keywords:** miRNAs, Tendon, Tendon injury, Tendon repair, Tendon healing

## Abstract

Tendon injury is one of the most common disorders of the musculoskeletal system, with a higher likelihood of occurrence in elderly individuals and athletes. In posthealing tendons, two undesirable consequences, tissue fibrosis and a reduction in mechanical properties, usually occur, resulting in an increased probability of rerupture or reinjury; thus, it is necessary to propose an appropriate treatment. Currently, most methods do not sufficiently modulate the tendon healing process and restore the function and structure of the injured tendon to those of a normal tendon, since there is still inadequate information about the effects of multiple cellular and other relevant signaling pathways on tendon healing and how the expression of their components is regulated. microRNAs are vital targets for promoting tendon repair and can modulate the expression of biological components in signaling pathways involved in various physiological and pathological responses. miRNAs are a type of noncoding ribonucleic acid essential for regulating processes such as cell proliferation, differentiation, migration and apoptosis; inflammatory responses; vascularization; fibrosis; and tissue repair. This article focuses on the biogenesis response of miRNAs while presenting their mechanisms in tendon healing with perspectives and suggestions.

## Introduction

**Fig. 1:**
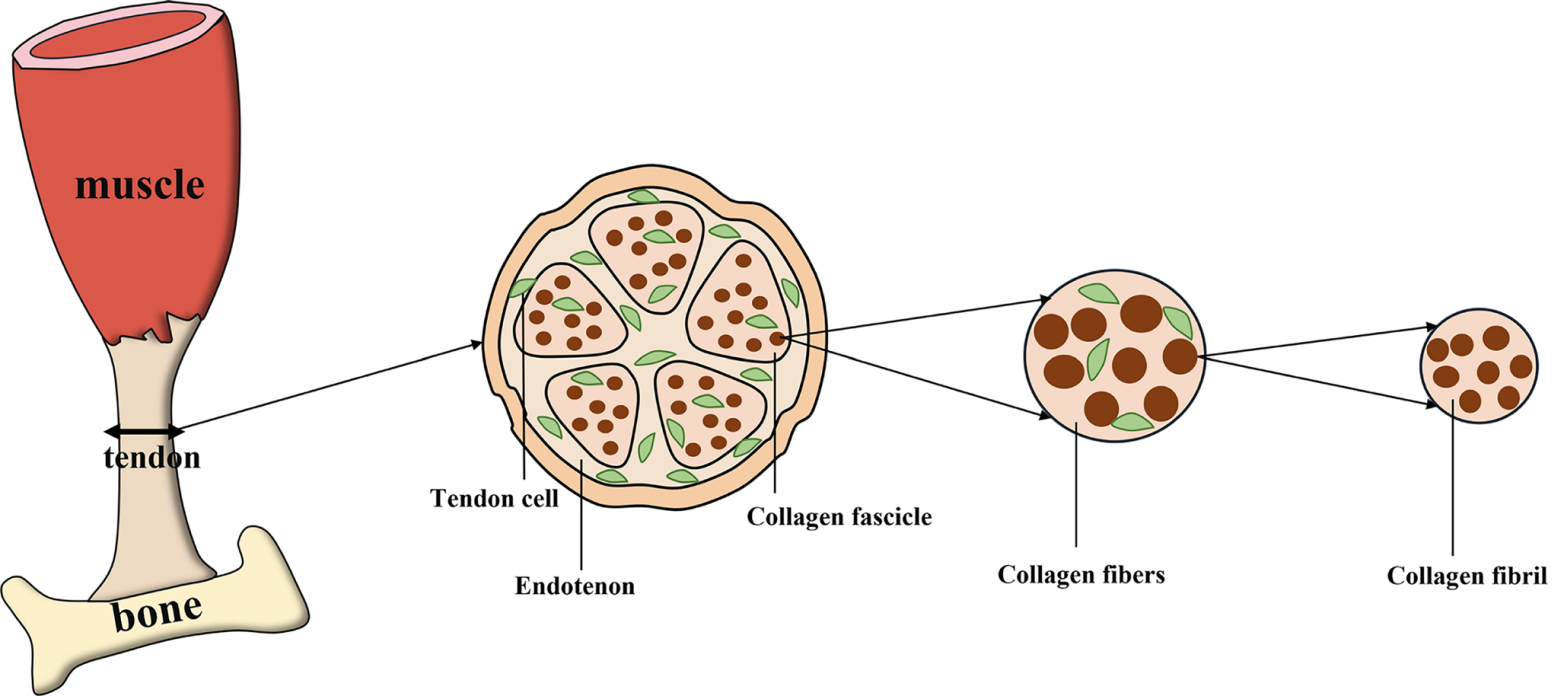
The structure of the tendon. Tendons mainly connect muscles to bones. The tendon is encased in a tendon sheath, which is designed to protect the tendon from damage and to reduce friction. The tendon sheath consists of an outer parietal layer (fibrous layer) and an inner visceral layer (synovial layer), which is divided into an inner synovial layer (peritenon) and an outer synovial layer (epitenon), with synovial fluid filling the area between the outer synovial layer and the fibrous layer. Multiple bundles of collagenous protofibrils converge to form collagen fibers. Collagen fibers are closely linked to form the collagen fascicle, which, together with tendon cells, forms a confined space called the endotenon

Tendons are connective tissues rich in collagen and tendon cells that play an important role in this aspect of the muscle-bone connection (Jin et al. 2022; Loiacono et al. 2019; Tsai et al. 2012). The fundamental roles of tendons include transmitting force and limiting muscle overload (Loiacono et al. 2019). Tendon injury, a common type of musculoskeletal disorder, is usually accompanied by pathological changes such as a disorganized collagen arrangement and increased amount of substrate material (Hast et al. 2014; Sakabe and Sakai 2011; Xu and Murrell 2008). The risk of tendon rupture increases gradually as the damage to the tendon becomes more severe, and most studies show that tendons heal slowly (Andarawis-Puri et al. 2015; Hast et al. 2014). The structure of the tendon is shown in Fig. 1.

The process of tendon healing typically consists of three overlapping periods: the inflammatory phase, the fibrotic phase, and the remodeling phase (Adabbo et al. 2016). Additionally, both intrinsic and extrinsic healing mechanisms are involved in each phase of tendon healing (Wu et al. 2017). Extrinsic healing occurs primarily as inflammatory cells move to the injured site and collagen synthesis is initiated, resulting in scar formation (Ding et al. 2021; Wu et al. 2017). Intrinsic healing, in contrast, is essentially the process by which tendon stem or progenitor cells (TSPCs) convene to facilitate tendon healing (Wu et al. 2017). Most therapies for tendon healing are usually evaluated by the symptomatic, structural, functional, and psychological aspects of the injury (Silbernagel et al. 2020). Although most therapies can restore homeostasis within the tendon, the complexity of the anatomical structure and biomechanical characteristics of the tendon are closely related to the therapeutic efficacy; thus, the process for determining the appropriate treatment modality still needs further refinement (Drew et al. 2014).

Epigenetic modifications occur primarily in response to changes in the tissue environment and regulate some cellular activities; these modifications include DNA methylation, histone methylation, acetylation, and miRNA expression (Tarnowski et al. 2022). miRNAs serve as an interesting therapeutic tool for altering some cellular functions and thus ameliorating some disease properties, as indicated by the observation that an individual miRNA can often interact with many target genes to further influence the related pathway or molecule, thus achieving a therapeutic effect, although this ability could also constitute a therapeutic disadvantage, since miRNAs target multiple genes may be more effective in restoring a certain segment of properties (Diener et al. 2022). In cancer, for example, miRNAs often act as tumor suppressors or oncogenic factors depending on their properties, meaning that they may have a dual role in cancer treatment based on either the suppression or activation of different factors, and perhaps the function of miRNAs in the process of tendon repair is not entirely effective (Menon et al. 2022). Currently, miRNAs are available as a relatively promising form of gene therapy and are often isolated from cells, tissues, tears, blood, and urine; they are used primarily to enhance tendon repair by attenuating certain degenerative changes and inflammatory responses. It has been shown that as molecules capable of regulating gene expression, some miRNAs may induce the pathological process of tendon injury, while the rest can exert reparative effects. In animal models, the sequences of miRNAs are not precisely complementary to those of their targets, implying that the miRNAs and targets are not fully regulated by each other (Ambros 2004). Overall, miRNAs play an important role in both the onset and prognosis of tendon injury, and they are involved mainly in regulating angiogenesis, ECM remodeling, tendon cell differentiation, and the inflammatory response to influence the process of tendon healing.

Although numerous studies have demonstrated the positive effects of miRNAs on tendon repair, their regulation, transport, and storage still need to be addressed. As Kabekkodu and Ding et al. found in their study, the transport of miRNAs is still problematic; exosomes, although they will be used as miRNA transport carriers, are not stable, and once a more consistent auxiliary carrier system is applied, the effect of tendon healing will be improved by more direct targeting of miRNAs to key genes (Ding et al. 2021; Kabekkodu et al. 2018). However, Lin et al. suggested that the detection of cancer cell-derived exosomal miRNAs (exo-miRNAs) can provide information for biopsies, suggesting that exosomes may be useful as vectors, although their role in tendon repair needs to be further explored (Lin et al. 2022). In addition, a large number of studies have shown that the improvement of biomechanical properties after tendon repair is still a difficult challenge in the field of tendon injury, and only a few research groups, such as Chatterjee et al. and Marqueti et al. have been able to show that the combined effect of TGF-β and mechanical loading can alter the expression of various miRNAs (Chatterjee et al. 2022; Marqueti et al. 2022). If miRNAs can constitute the main therapeutic breakthrough, can they provide an important basis for the recovery of mechanical properties? This ability needs to be proven by further studies.

Our article focuses mainly on the more well-studied miRNAs to describe their mechanisms of action; for example, we focus on the miR-29 family, mainly miR29a and miR29b, which are closely related to collagen synthesis, regulation of BMP levels, tendon adhesion and other repair processes, and we do not yet know whether other miR29 family members also play a role in tendon repair (Millar et al. 2015; Watts et al. 2017). For example, Horita et al. showed the partial role of the miR29 family in diseases such as osteoarthritis and osteoporosis-associated immune dysfunction; thus, it is possible that some members can also play a role in tendon repair (Horita et al. 2021). Therefore, the purpose of this review is not only to elaborate the in-depth mechanisms of various miRNAs in repairing tendon injury but also to show examples of their application in other disorders, such as tendon–bone repair and muscle injury, and to provide a preclinical basis and new therapeutic insights for the treatment of tendon injury.

## Explanation of the anatomy and biomechanical function of tendons

The tendon is actually a complex physiological system consisting mainly of a fibrous collagen core (tendon cells and associated collagen components) forming an intrinsic compartment, while the extrinsic compartment is composed of synovial-like tissue; these two compartments interact with each other and are interspersed with some blood vessels and nerves, with tendon repair closely related to the activity of both compartments and mainly including changes in tendon fibroblasts, blood vessels and collagen fibers (Snedeker and Foolen 2017). Various tendon tissues, thanks to their differences in structure and components, are essential for the complexity of tendon anatomy, and the effectiveness of a wide range of treatments may yield diverse results when applied to each structure. For example, the tissue of the Achilles tendon, the most well-studied tendon, forms fibrovascular scars in healed Achilles tendons, resulting in weaker mechanical properties than those of intact tendons (Shapiro et al. 2015). In addition, Lehner et al. identified a blood-tendon barrier (which is a functional endothelial barrier), a structure that limits the entry of blood-derived molecules into the surrounding tendon tissue, which results in less vasculogenesis in the early stages of repair in Achilles tendon tissue, which contains a small number of blood vessels, ultimately leading to poor repair (Lehner et al. 2016). The inflammatory state of rotator cuff tendon tissue has been extensively studied and is usually due to the infiltration of a specific cell type, namely, adipocytes, which further slows the tendon healing process (Giordano et al. 2020; Thankam et al. 2019a, b). Because of the structural peculiarities of the shoulder, lesions of the rotator cuff tendon are normally accompanied by fibrosis of the subacromial capsule, with robust angiogenesis (Ko et al. 2019). Although the patellar tendon is superficial and well stripped, it is surrounded by the collateral and cruciate ligaments, making its healing difficult, especially in the presence of reduced cell numbers and oxygen supply to the tendon core (Kia et al. 2018; Notermans et al. 2021). The different structures of the various tendon tissues make the repair process more difficult.

Collagen is the most abundant protein in vertebrates, and collagen fibers, which are the primary components that provide support for connective tissue and tissue morphology, coordinate physiological phenomena such as angiogenesis, wound healing, and biomineralization in bone, tendon, and skin tissues (San Antonio et al. 2020; Wu et al. 2022). The basic unit of force transmission in tendons is mainly the collagen fiber, comprised mainly of three chains intertwined to form a spiral structure and crosslinked to create the fixed structure of the tendon, thus giving the tendon tissue an increased mechanical strength (Ding et al. 2021; Wu et al. 2022). In addition, some studies in animal models have proven that the activity of early collagen fibers directly affects the mechanical and structural properties of tendon tissue, implying that changes in collagen fibers have positive implications for the alteration of biomechanical properties; however, the variety and complexity of collagen fiber activity and the number of biological factors involved suggest that restoring the mechanical properties of tendons remains a major challenge (Freedman et al. 2016). Professor Holmes, however, suggests a more important phenomenon, namely, that these structures can be generated mainly through a tissue of fibroblasts matched to the embryonic tendon, further demonstrating that there is some interaction between the skeleton of the cells and the extracellular matrix—a phenomenon also referred to as mechanical oscillation—and that this activity is closely related to the sensing and stretching of the tendon tissue and could perhaps be an important link in tuning the mechanical properties of the tendon (Holmes et al. 2018). Additionally, the maintenance of mechanical properties is associated with the circadian rhythm of collagen fibers, a surprising finding. For example, Kadler et al. suggest that circadian rhythms regulate the homeostasis of tendon tissue, with collagen production and secretion being the primary processes affected, which in turn affects ECM remodeling, endoplasmic reticulum homeostasis, and the rest-activity cycle and drives tissue homeostasis; thus, regulation of the peripheral clock in conjunction with circadian rhythms may serve as a target for alleviating tendon injury and thus regulating the reduced performance in the studied model (Yeung and Kadler 2019).

## The roles and functions of miRNAs in tendon repair

### Basic information about tendon repair

As described in the literature, the process of tendon repair is usually divided into three phases. In the first phase, the inflammatory response phase, which lasts for approximately 48 h, large amounts of cytokines and growth factors are released after partial tendon tissue injury, where factors associated with increased vasoactivity come into play, maintaining the most important feature of early repair (Lyu et al. 2022; Sakabe and Sakai 2011). The proliferative phase, however, is dominated by the migration of tendon fibroblasts and epithelial cells to the site of injury, followed by the occurrence and development in conjunction with the synthesis of collagen, resulting in the deposition of extracellular matrix (Guerra et al. 2013; Tsai et al. 2012). Eventually, during the remodeling phase, a large number of matrix metalloproteinases come into play, among which MMP14 was shown by Lu et al. to construct a natural tissue-like ecotone that is quite conducive to tendon healing. In addition, a small number of resident mast cells and macrophages also come into play during this phase, culminating in increased type 1 collagen synthesis and promoting tendon healing (Koh and DiPietro 2011; Lu et al. 2021).

However, the process of tendon repair is somewhat different from that of some other tissues. For example, the healing of skin tissues usually revolves around autophagy, and although the skin tissue healing process can be divided into three phases, there are some differences in the molecules and mechanisms involved, for example, the healing of ulcer wounds caused by diabetes is slowed by the regulation of autophagy by stem cells (Ren et al. 2022). In addition, tendon healing and tendon–bone healing are similar processes, especially in rotator cuff injuries; thus, it would be judicious to investigate strategies for the repair of other tissues, such as the use of drugs (Ricofeminone23, Tamoxifen3), stem cell exosomes and small molecule inhibitors (Huang et al. 2020; Wang et al. 2020), to improve tendon healing in future studies.

Tendon repair still faces many challenges. Restoration of tendon mechanical properties is one of the most difficult tasks; for example, as Snedeker et al. suggested, although both collagen and fibronectin can be used to treat tendon and ligament healing and occasionally show good prognosis in preclinical studies, it is still a great challenge to restore tendon biomechanical properties (Snedeker and Foolen 2017). Our group has used ultrasound to treat rats with tendon injuries, and after treatment, we examined changes in mechanical properties in both the model and treated groups and found little change, implying that there is still great difficulty in finding a treatment modality that can enhance the mechanical properties of the repaired tendon. In addition, since tendon repair involves many factors, such as the immune system, chemical mediators, mechanical stimuli, and biological factors and cytokines, if these factors are not in homeostasis, the healing response may fail; thus, means to determine the stability of these factors need to be further improved (Chisari et al. 2020).

### Biological information about MicroRNAs

In 1993, researchers discovered the first miRNA, and 7 years later, the existence of the miRNA-let7 family was proven. To date, we have studied miRNAs for more than 20 years and discovered most of their biological properties (Ding et al. 2021). miRNAs, which typically repress gene expression, can regulate nearly half of the genes encoded in the human genome (Dakin 2017; Dubin et al. 2018; Watts et al. 2017). At present, miRNAs are categorized clinically into two groups: intracellular miRNAs and extracellular miRNAs (also referred to as circulating miRNAs) (Ding et al. 2021). In general, miRNAs bind to the 3′ untranslated region (UTR) of a target gene to downregulate the expression of the target gene, thus inhibiting the synthesis of its messenger RNA (Dubin et al. 2018; Liu et al. 2019).

**Fig. 2:**
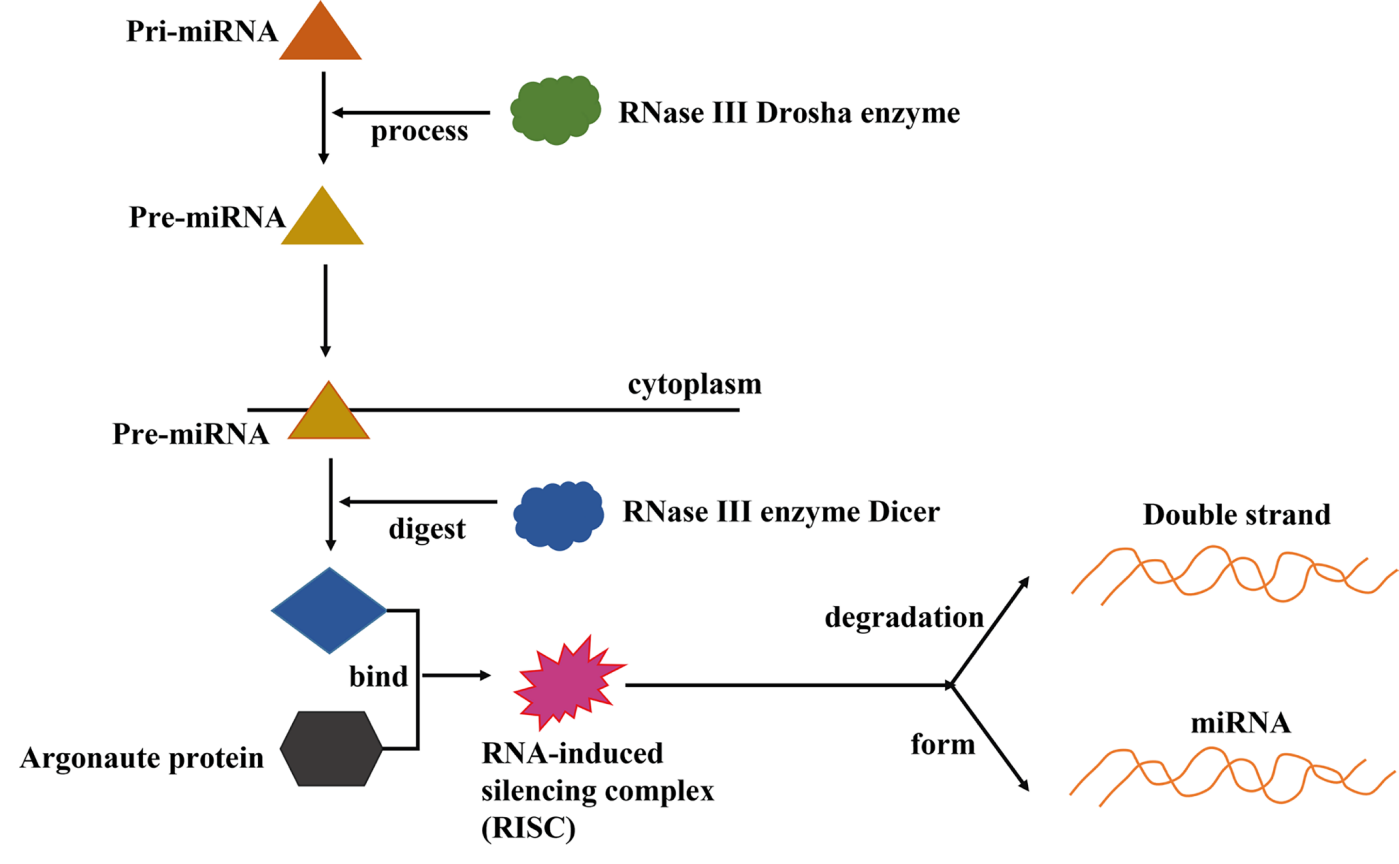
Process of miRNA biogenesis

The biogenesis of miRNAs involves multiple processes, and it has been revealed that in most cases, miRNAs are intimately linked to the transcriptional activity of ribonucleic acid polymerase II; however, transcription by Pol III can also contribute to the synthesis of a small fraction of miRNAs (Ding et al. 2021; Lam et al. 2020). Primarily, when the RNAse III enzyme Drosha acts on primary transcripts (pri-miRNAs), substantial amounts of precursor miRNAs (pre-miRNAs) are generated and are transported to the cytoplasm for the next step (Lam et al. 2020; Tiwari et al. 2018). Subsequently, the pre-miRNA is degraded by the RNAseIII enzyme Dicer, and then, one of the chains binds to the Dicer, TAR RNA binding protein **(**TRBP), and Argonaute2 proteins to form the RNA-inducible silencing complex (RISC). Additionally, Argonaute2 proteins cleave the rest of the chain, eventually forming the mature miRNA (Sunwoo et al. 2020; Tiwari et al. 2018). The process of miRNA biogenesis is illustrated in Fig. 2.

### MicroRNAs regulate tendon repair

**Fig. 3:**
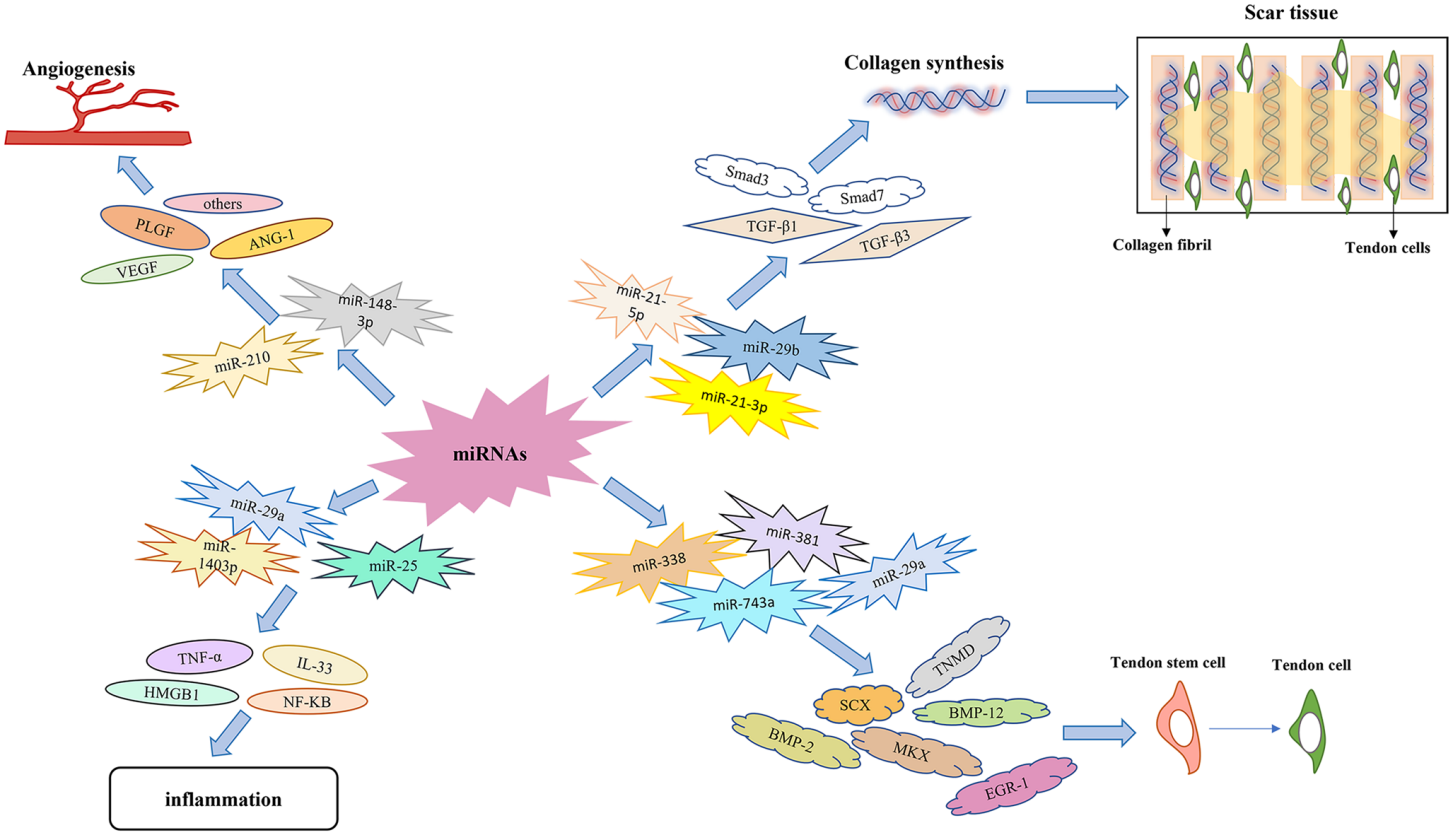
Mechanisms of miRNAs in the process of tendon healing

The process of tissue healing often requires the transmission of messages between cells. The results of numerous in vivo trials indicate that exosomes can act as transmitters of cell-to-cell communication, leading to the delivery of miRNAs, and this is the predominant mode of miRNA transport to the site of injury (Bjorge et al. 2017; Valadi et al. 2007). The exosome-mediated transfer of miRNAs, such as miR-17, miR-18, and miR-375, has been proven to function in angiogenesis and tumor formation; however, this mechanism is not applicable for the treatment of all tendons (Lam et al. 2020; Valadi et al. 2007). However, miRNAs are also able to modulate certain key signaling pathways, such as the Wnt and mTOR pathways (Yao et al. 2021). It was shown that miRNA-155 could upregulate the expression of inflammatory genes in a model of endotoxin injury, while miRNA-146a could only exert an inhibitory effect (Bjorge et al. 2017). Progress is being made in the use of miRNAs as a gene therapy, suggesting that such treatment positively impacts tendon healing, usually by reducing inflammatory and degenerative changes (Ilaltdinov et al. 2021). The mechanisms of miRNAs in the tendon repair process are shown in Fig. 3.

After healing is initiated, miRNAs are transported to the site of injury, thus activating exogenous and endogenous repair mechanisms for tendon healing, which accelerates neurovascular repair and modulates the development of adverse effects such as tissue disorders and reductions in mechanical properties (Snedeker and Foolen 2017; Zhou et al. 2020). In general, the endogenous mechanism mainly involves the proliferation of tendon cells, which is the most crucial process for increasing tendon strength, while the exogenous mechanism tends to induce the adverse effect of tendon adhesion, including the entry of fibroblasts and inflammatory factors into the site of injury (Wellings et al. 2021; Zhou et al. 2020). Additionally, the typical mechanisms associated with tendon healing are cell proliferation and differentiation, angiogenesis, alteration of the inflammatory response, and collagen synthesis (Ding et al. 2021; Liu et al. 2021b). The miRNAs involved in the process of tendon healing are listed in Table 1.

**Table 1:** miRNAs involved in the tendon healing process

**miRNA**	**Targets**	**Models**	**Functions**	**References**
**miR-210**	VEGF Type I collagen↑ FGF-2↑	A rat Achilles tendon injury model	Promotes angiogenesis	(Liu et al. 2021a, b)
**miR-148-3p**	thrombospondin 4↑ Krüppel-like factor 6↓	**/**	Boosts angiogenesis in endothelial cells	(Giordano et al. 2020)
**miR-21-5p**	Smad7↓	A model of tendon injury in mice	Facilitates tendon cell proliferation and differentiation while affecting the degree of tendon fibrosis	(Cui et al. 2019)
**miR-21-3p**	Type III collagen↓ α-SMA↓	**/**	Closely related to tendon fibrosis and adhesions	(Harrell et al. 2019)
**miR-29b**	TGF-β/Smad3↓ Type I collagen↓	Animal models of common tendon injuries	Prevents adhesions after tendon injury surgery	(Dubin et al. 2018; Zhou et al. 2020)
**miR-29a**	Type III collagen↓ BMP-2↓ BMP-12↓	Patients with clinical rotator cuff tears	Regulates the accumulation of fibrotic matrix in tendon cells and collagen production in pathological tissues Maintains the stability of tendon cell differentiation	(Howell et al. 2017; Ko et al. 2019)
**miR-25**	TNF-α↓ HMGB1↓	Degenerative rotator cuff tendinopathy	Involved in the regulation of inflammatory response	(Plachel et al. 2020)
**miR-1403p**	NF-kB↓	Degenerative rotator cuff tendinopathy	Attenuates the inflammatory response and reduces the degeneration of tendons	(Plachel et al. 2020)
**miR-19**	JAK-STAT↑ IL-6↑ MMP-3↑	Degenerative rotator cuff tendinopathy	Attenuates the inflammatory response	(Plachel et al. 2020)
**miR-378a**	SCX↓ Mkx↓ Type I collagen↓	miR-378a knock-in transgenic mice	Inhibits tendon differentiation and reduces the efficiency of tendon healing	(Liu et al. 2019)
**miR-124-3p**	EGR-1↓	**/**	Anti-miR-124 may serve as an effective substance to promote tendon differentiation	(Giordano et al. 2020)

### miRNAs promote angiogenesis

The initial stage of tendon healing, the inflammatory stage, is accompanied by damage to the original vascular system and the acceleration of metabolism (Li et al. 2021). At the same time, there is progressive hypoxia in the local tissues, which results in three consequences: angiogenesis, inflammation, and oxidative stress (Li et al. 2021; Sakabe and Sakai 2011). Increased vascular permeability and the formation of a large number of vascular systems imply the activity of angiogenesis, which is the most evident feature of the inflammatory phase (Sakabe and Sakai 2011; Sharma and Maffulli 2006; Xu and Murrell 2008). Several growth factors are closely associated with angiogenesis, namely, vascular endothelial growth factor (VEGF), angiopoietin-1 (ANG-1), and placental growth factor (PLGF), and when they are activated, their expression gradually increases, which means that the permeability of blood vessels increases and the program of angiogenesis is initiated (Tiwari et al. 2018).

Angiogenesis usually involves the following six steps: (1) promotion of endothelial cell activation, (2) enzymatic degradation of the capillary basement membrane, (3) proliferation and transfer of endothelial cells, (4) appearance of endothelial cell tubes, (5) fusion and modification of blood vessels, and (6) formation of a large capillary network (Tiwari et al. 2018). It has been proposed that a variety of miRNAs are involved in mediating the angiogenic process, and the most effective miRNA is miR-210, which mainly promotes the synthesis of type 1 collagen and upregulates the expression of VEGF (Cui et al. 2019; Lie atal. 2021b; Watts et al. 2017). In the animal model established by Liu et al., an increase in the diameter of collagen fibers and an expansion of capillary density were observed after miR-210 was injected into the Achilles tendons of the rats (Liu et al. 2021b). Moreover, angiogenesis gradually ceases during the proliferative phase of tendon repair, suggesting that miR-210 acts only during the inflammatory phase (Giordano et al. 2020; Liu et al 2021b). MiR-148-3p has also been suggested to be important in promoting angiogenesis during tendon healing. It promotes angiogenesis in the pathological process of tissue tendon injury mainly by upregulating the gene expression of thrombospondin-4 while suppressing the expression of Krüppel-like factor 6 (KLF6) (Giordano et al. 2020). However, there are relatively limited studies on the proangiogenic effect of miR-148-3p; hence, further elucidation of its mechanism of action is required. Although angiogenesis is important for tendon healing, excessive formation of blood vessels may have adverse consequences for tendon healing.

Currently, exosomes are used as a cell-free therapeutic modality to alleviate tendon injury, and they rely heavily on miRNAs to exert their effects. Heo et al. demonstrated that miR-132 and miR-146a released from exosomes can regulate the expression of genes related to angiogenesis, with miR-132 able to bind to thrombospondin-1 (THBS1), an antiangiogenic mRNA, while miR-146a targets vasopressor-1 (VASH1) and thus enhances endothelial progenitor cell function, but whether these two miRNAs can induce tendon injury repair remains to be demonstrated in numerous experiments (Heo and Kim 2022). In parallel to the role of miRNAs in facilitating angiogenesis during tendon healing, extensive investigations are proving the presence of miRNAs in diseases such as colon, stomach, and breast cancer (Tiwari et al. 2018). Therefore, miRNAs possess the ability to target different genes, which could be a prerequisite for or evidence to support the development of antiangiogenic drugs.

### miRNAs modulate tendon adhesions

The ability for the function of an injured tendon to be restored by its regenerative capacity often results in the formation of mechanically deficient scar tissue in the area of injury, which is the most challenging complication of tendon repair. Scar tissue formation is usually closely related to collagen synthesis, and the predominant type of collagen is type I; however, when the scar tissue content is excessively high, the tendon adheres substantially (Andarawis-Puri et al. 2015; Wu et al. 2017; Zhang et al. 2021). Scar tissue is converted mainly from fibrous tissue, and its formation starts after the remodeling phase and lasts for a relatively long period of time (de la Durantaye et al. 2014; Sharma and Maffulli 2006). Due to the two mechanisms of tendon healing (external and internal), the roles played by various factors are distinct. However, external healing commonly results in the formation of scar tissue that damages the mechanical properties and impairs the gliding ability of the tendon, and although preclinical studies have introduced biomaterial techniques to ameliorate this negative phenomenon, few modalities have led to a fully satisfactory prognosis (Liu et al 2021b; Sharma and Maffulli 2006).

Transforming growth factor-β (TGF-β), the growth factor most closely associated with the scar formation process, converts fibroblasts into α-smooth muscle actin (α-SMA)-positive myofibroblasts, indicating that the main feature of tendon adhesions is ECM synthesis (Chen et al. 2009; Li et al. 2021; Yao et al. 2020). TGF-β has three isoforms: TGF-β1 and TGF-β2, which mainly regulate scar formation and fibrosis in tissues, and TGF-β3, which has functions opposite those of TGF-β1 and TGF-β2. Among the TGF-β isoforms, TGF-β1 mainly activates Smad3, which is the most dominant signaling pathway in promoting tendon injury repair (Jiang et al. 2021). TGF-β3, however, targets Smad7, downregulates Smad3, and inhibits the function of TGF-β1; TGF-β3 can be used as a therapeutic tool to achieve scarless tendon healing, thus reducing the formation of tendon adhesions (Jiang et al. 2021). Whereas CTGF, a downstream mediator of TGF-β-induced tendon fibrosis, is usually closely associated with ECM formation, the experiments of Chen et al. demonstrated that certain miRNAs were able to silence the expression of TGF-β but had a lesser effect on CTGF and that type I collagen synthesis was not decreased, implying that CTGF could also be used as a target of miRNAs for the treatment of tendon fibrosis; however, the exact miRNAs involved need to be further identified (Chen et al. 2009).

Several types of miRNAs can be involved in collagen synthesis. Among them, Rutnam et al. demonstrated that during mammary gland, lung epithelial cell, and lung fibrogenesis, miR-21 can regulate type I collagen synthesis (Rutnam et al. 2013). In the process of tendon repair, different miR-21 family members can exert their effects by regulating different molecules. For example, miR-21-5p carried in exosomes of bone marrow macrophages can target Smad7, thus promoting tendon cell proliferation and differentiation (Ding et al. 2021; Lu et al. 2021). Human umbilical cord stem cell-derived exosomes, on the other hand, can carry large amounts of miR-21-3p to modulate p65 activity (Ding et al. 2021; Lu et al. 2021; Yao et al. 2020). However, according to the experiments of Lu et al., tendon healing is extremely effective when miR-21-3p activates TGF-β1 while regulating type I collagen expression; thus, inhibition of TGF-β1 alone may not facilitate tendon healing, and numerous studies are still needed to verify whether TGF-β2 and TGF-β3 affect tendon adhesion formation (Dubin et al. 2018; Liu et al. 2021b). Furthermore, miR-29b has been shown to reduce the extent of tendon adhesion by regulating the TGF-β/Smad3 signaling pathway; however, the miR-29b binding site in collagen I is located in the 3'UTR, unlike its binding site in TGF-β1, implying differences in the binding sites between an miRNA and its targets (Ding et al. 2021; Dubin et al. 2018; Watts et al. 2017). In general, miR-29b mainly inhibits the TGF-β/Smad3 signaling pathway, thus controlling the proliferation of fibroblasts and ultimately improving the tendon healing effect (Liu et al. 2021b). When miR-29b is combined with certain drugs, it may decrease tendon adhesions while ensuring the mechanical properties of tendons (Zhou et al. 2020). For example, Zhou et al. demonstrated that miR-29b inhibitors have better repair effects when combined with tanshinone IIA to treat tendon injuries; thus, some combinations of herbal medicines and miRNAs are potential therapeutic approaches for reducing tendon adhesions, constituting a viable approach to tendon repair, but experiments are needed to demonstrate whether there are negative effects (Zhou et al. 2020).

### miRNAs decrease the inflammatory response

The inflammatory response after tendon injury usually lasts for 48 h and entails alterations in growth factors and cytokines, caused mainly by signaling-specific or gene-specific cascade mechanisms, in which large amounts of inflammatory factors disrupt physiological homeostasis and stimulate the site of injury, ultimately affecting tissue regeneration and healing (Lopes Silva et al. 2020; Sakabe and Sakai 2011; Finosh G. Thankam et al. 2019a, b). Among the two healing mechanisms of tendon repair, the external healing process starts earlier, indicating that a large number of inflammatory cells move to the site of injury and initiate the inflammatory response (Sakabe and Sakai 2011; Wu et al. 2017). The levels of certain metabolic substances, such as glutamate and pyruvate, are increased in injured tendons compared to normal tendons, implying that the metabolic processing of these substances is more active early in the course of tendon injury, while the glycolytic process remains active for longer, during almost the entire process of tendon repair, suggesting that the inflammatory response can regulate the function of various cells during the healing process (Ackerman et al. 2021; Klatte-Schulz et al. 2018). Therefore, altering the metabolic processing of various substrates in the inflammatory response can also be a strategy for the treatment of tendon injuries.

**Fig. 4:**
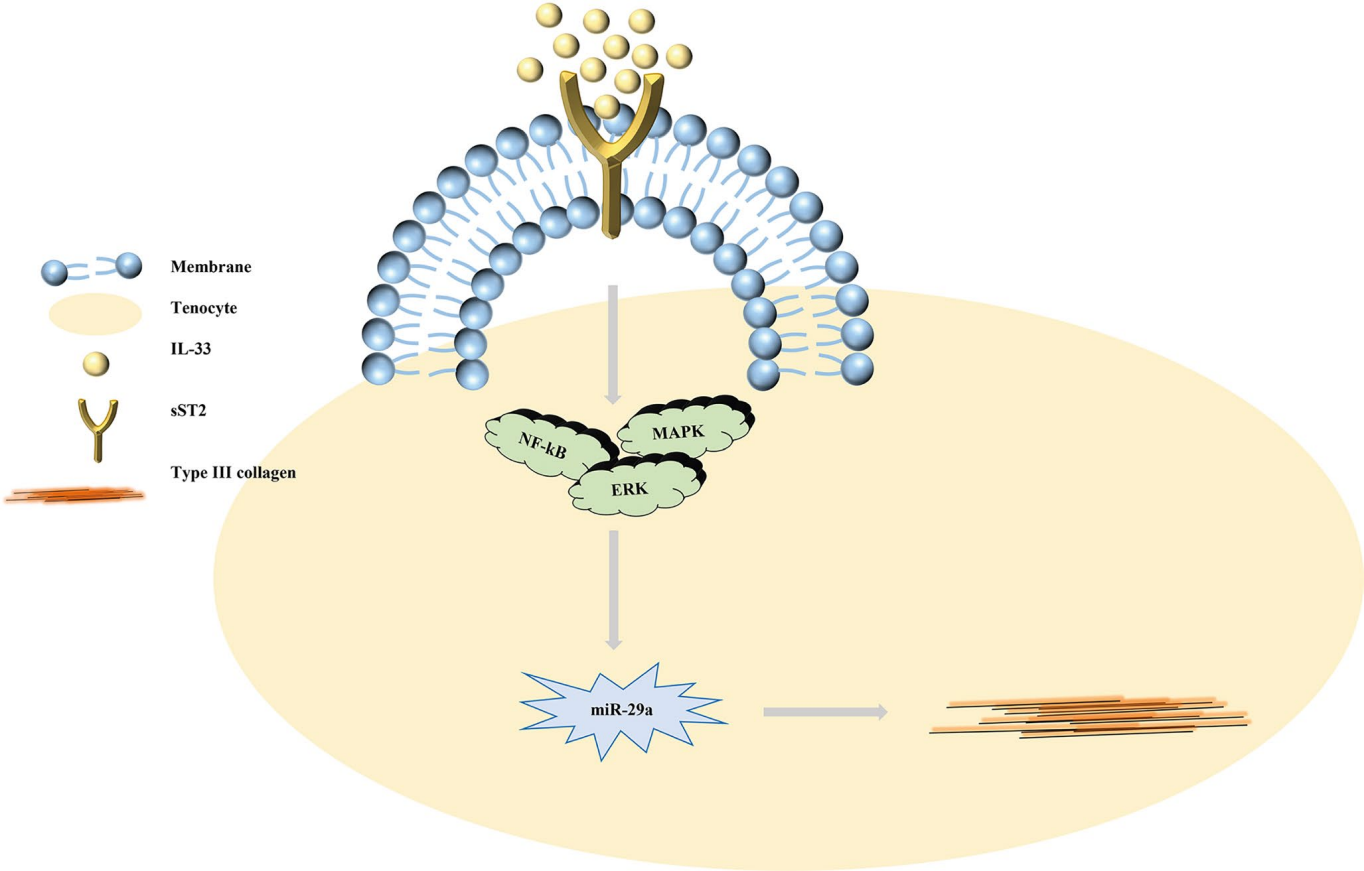
miR-29a affects the inflammatory response during the early phase of tendon repair. IL-33 binds to ST2, thereby promoting downstream phosphorylation of NF-KB, further inhibiting miR-29a production and ultimately contributing to an increase in type III collagen production

In general, miRNAs can act as ligands to bind to several immune receptors, leading to the regulation of inflammatory responses (Ding et al. 2021). According to the results in the tendinopathy model established by Millar et al., the imbalance in matrix homeostasis induced in the early stages of tendon injury is caused by interleukin-33 (IL-33) secreted by tendon cells, which activates ST2 receptors and thus increases type III collagen synthesis, the most typical manifestation of the inflammatory phase (Liu et al. 2021b; Millar et al. 2015; Xiao et al. 2019). Based on the role of IL-33 in various inflammatory diseases, miR-29a is involved in a regulatory link in which tendon cells secrete large amounts of IL-33; this IL-33 then binds to some isoforms of ST2, promoting NF-KB phosphorylation, which inhibits miR-29a expression and promotes type III collagen synthesis, ultimately regulating the inflammatory response. miR-29a tends to also affect another mechanism of this pathway, which may also indicate IL-33 as a target in tendon injury, with elevated miR-29a triggering a surge in sST2 expression and thus reducing IL-33 levels in a feedback-regulated manner (Liu et al. 2021b; Millar et al. 2015). In addition, Ko et al. demonstrated that certain pathological manifestations, such as swelling and a high degree of fibrosis, are reduced when miR-29a is overexpressed in rotator cuff injury (Ko et al. 2019). Overall, miR-29a can alleviate the inflammatory response because of its targeted silencing of the type III collagen gene (Liu et al. 2021b). The mechanism by which miRNA-29a regulates the inflammatory response is shown in Fig. 4.

In addition to miR-29a, the most critical marker for regulation of the inflammatory response, other miRNAs are being researched; however, there are still doubts about their mechanisms of action that need to be resolved. In degenerative rotator cuff tendinopathy, miR-25 is involved in the regulation of the inflammatory response and mainly suppresses the expression of tumor necrosis factor (TNF-α) and high mobility group box 1 (HMGB1), while whether miR-25 plays the same role in other tendon injuries remains questionable (Mosca et al. 2017; Plachel et al. 2020). The NF-KB signaling pathway is the most typical signaling pathway involved in mediating the inflammatory response in tendon injury (Chen et al. 2017), and it is usually negatively regulated by miR-1403p, a miRNA that suppresses the expression of nuclear receptor coactivator 1 (NCOA1) and nuclear receptor interaction protein (NRIP); thus, it is possible to attenuate the inflammatory response by upregulating miR-1403p (Plachel et al. 2020). In addition to the involvement of the NF-kB signaling pathway in the regulation of inflammatory responses, the expression of JAK-STAT signaling pathway regulators also affects the release of inflammatory factors, and Plachel et al. showed that miR-19 can negatively regulate the release of these factors, while inhibiting the synthesis of miR-19 enhances the increase in the production of inflammatory cytokines (IL-6 and MMP-3), resulting in an inflammatory response, which means that upregulation of miR-19 can alleviate the inflammatory response (Plachel et al. 2020).

Generally, the expression of hypoxia-inducible factor (HIF-α) in tissues after tendon injury is increased, which promotes the expression of NF-kB signaling pathway components and regulate extracellular signal-regulated kinase 1/2 (ERK1/2) in the MAPK signaling pathway, P38, and the phosphorylation level of JNK, resulting in an inflammatory response (Jiang et al. 2021; Jiao et al. 2022). Li et al. demonstrated that in the tubulointerstitium, HIF-1α can increase the level of exosomal miR-23a in hypoxic tubular epithelial cells, thereby activating macrophages, implying that targeting HIF-1α to regulate miRNA expression can be used as a way to treat tendon injuries (Jiao et al. 2022). In summary, the inflammatory response, the most robust response to tendon repair, typically involves changes in multiple biological factors, and we need to further investigate the differences in therapeutic targets and explore the roles miRNAs play in these differences.

### miRNAs mediate tendon differentiation

In general, tendon-derived differentiation of tendon stem/progenitor cells is affected by distinct miRNAs in a manner dependent on their regulation of the expression of diverse growth factors and related protein genes. The process of tendon stem/progenitor cell differentiation is essential for maintaining intratendon homeostasis and is typically regulated by three biological factors, namely, helix-loop-helix transcription factor (SCX), Mohawk (MKX), and early growth response 1 (EGR1), whose expression can be influenced by tendon regulatory proteins (Nourissat et al. 2015). Few in vitro experiments have been able to fully elucidate the link among these three growth factors, but ectopic expression of Mkx upregulates the gene expression of SCX in mouse tendon stem cells, and whether this can also occur in the context of tendon injury in humans is inconclusive (Nourissat et al. 2015).

A specific marker involved in tendon development and maturation is SCX, which not only stimulates tendon-derived differentiation of tendon stem/progenitor cells but also activates the expression of TNMD transcription factors in a tendon spectrum-dependent manner. The TNMD gene is essential for tendon maturation and tendon stem cell renewal; thus, the lack of SCX during repair leads to an incomplete tendon differentiation process, which affects the functional and structural restoration of damaged tendons (Liu et al. 2021a; Shi et al. 2019). Recent evidence suggests that under excessive tendon loading, the expression of three miRNAs that bind to SCX, namely, miRNA-338, miR-381, and miR-743a, is suppressed, thus affecting tendon differentiation and adversely influencing the outcome of tendon repair; however, the role of these miRNAs has not been fully validated (Mendias et al. 2012). The most extensive pathway involved in the process of tendon maturation and development is the TGF-β signaling pathway, which activates SCX during the initial stages of tendon stem cell differentiation and, as the tendon matures, can activate the expression of Mkx and eventually lead to tendon repair (Liu et al. 2019). Liu et al. demonstrated that miR-378a can inhibit the expression of SCX and Mkx because miR-378a can bind to TGF-β2 and inhibit its expression, thereby reducing the synthesis of collagen type I, which means that miR-378a can degrade collagen through degradation of the ECM, inhibit tendon differentiation and reduce the efficiency of tendon healing (Giordano et al. 2020; Liu et al. 2019). Furthermore, early growth response factor-1 (EGR-1) is also involved in mediating tendon differentiation. Wang et al. suggested that miR-124-3p binds to EGR-1 and inhibits its expression, thereby reducing the efficiency of tendon differentiation and decreasing collagen synthesis (Wang et al. 2016). Dubin et al. proved that miR-140 is also able to regulate the differentiation capacity of TSPCs, mainly by inhibiting the expression of Pin1, which is responsible for accelerating the senescence of tendon stem cells (Dubin et al. 2018). Moreover, ROCK1, a member of the serine/acid kinase family, mainly activates the expression of actin filaments while regulating cell differentiation, and its expression is suppressed by miR-135a, which inhibits tendon differentiation and migration; however, miR-135a has also been shown to play different roles in aged stem cells at different stages, a finding that still needs further proof (Ding et al. 2021; Dubin et al. 2018). The role of miRNA-135 was also demonstrated by Omoto et al., who used Dicer knockout mice to study the effect of miRNA-135 on the gait of mice and showed that this miRNA is related to the formation of Achilles tendon-derived fibroblasts (Omoto et al. 2022).

**Fig. 5:**
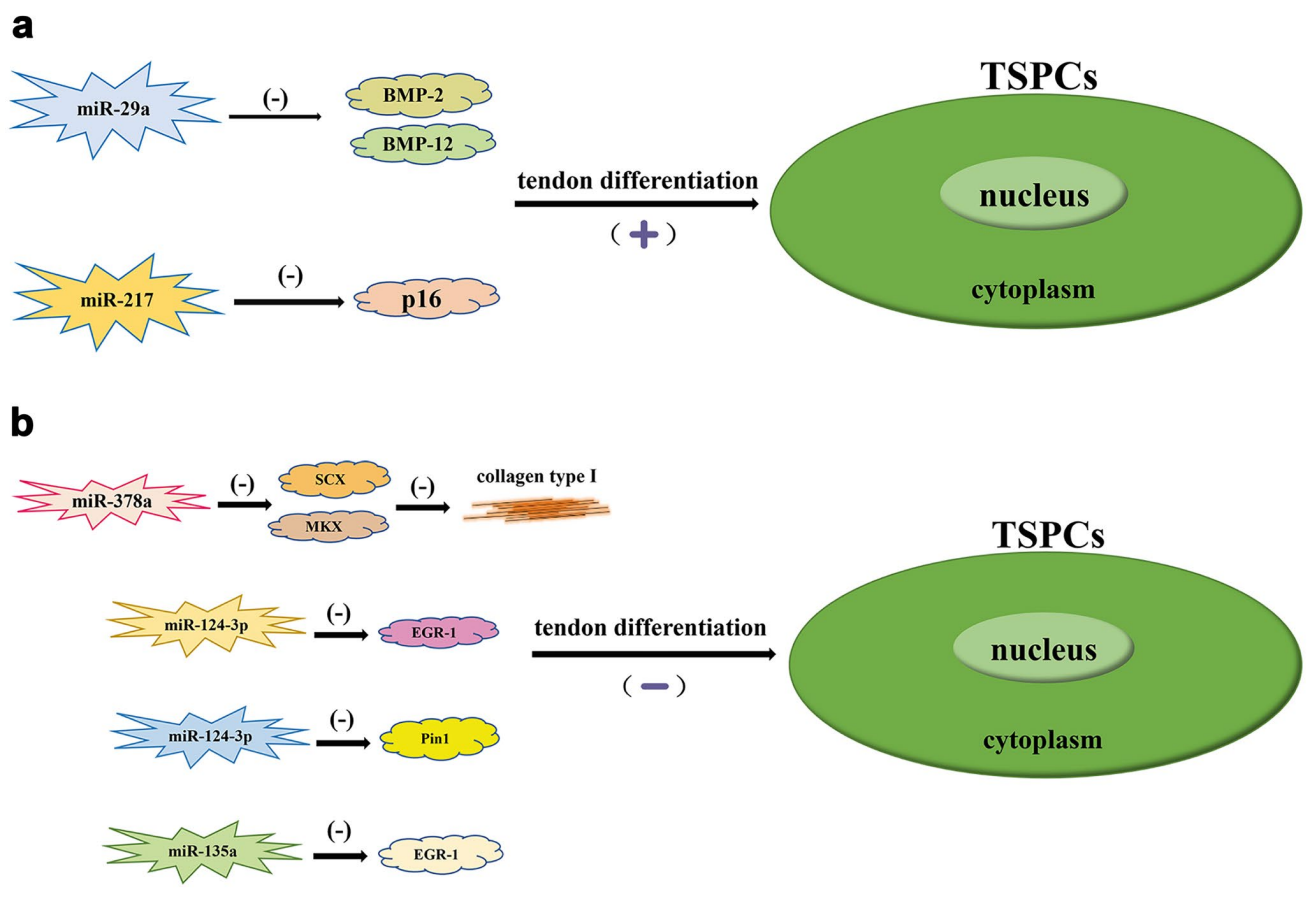
Various miRNAs modulating tendon differentiation

There are six miRNAs/miRNA families involved in the pathological phenomenon of tendon injury, namely, the let-7 family, miR-7a, miR-22, miR-26a/b, and miR-29a, which can regulate bone morphogenetic protein (BMP); however, the specific regulatory mechanisms of these miRNAs/miRNA families has not been elucidated (Liu et al. 2021b). BMP signaling can also function in tendon repair, and although it normally induces cartilage differentiation, individual BMPs, such as BMP-12, 13, and 14, can promote tendon differentiation behavior (Howell et al. 2017). Xiao et al. revealed that miR-29a was able to reduce the expression of BMP-2 and BMP-12, thus maintaining the stability of tendon cell differentiation (Xiao et al. 2019). This is due to the ability of BMP-2 to inhibit the mRNA expression of Scx in C2C12 myogenic cells, chondrocyte-like TC6 cells, and tendon-derived stem cells (Liu et al. 2014). When TSPCs become senescent, they gradually lose their differentiation ability, and the expression of the senescence marker p16, which is a direct target of miR-217, is significantly upregulated, implying that miR-217 could also be involved in regulating the process of tendon differentiation (Ding et al. 2021). The miRNAs regulating the differentiation of tendon stem/progenitor cells are shown in Fig. 5.

## Conclusion and perspectives

It is well known that tendon repair is a complex process whereby the structure and function of the repaired tendon is not completely restored. In contrast, miRNAs, as a promising therapeutic modality, can regulate the expression of various genes during tendon repair, thus affecting tendon development and functional recovery, but some miRNAs can also act as negatively regulated biomarkers, functioning as targets for tendon repair (Plachel et al. 2020). Based on numerous studies ins animal models, tendon healing is improved when the expression of certain miRNAs is upregulated or downregulated, but almost all of these experiments are preclinical studies, and the tendinopathy models do not fully mimic the extent of human tendon injury; thus, whether these miRNAs can affect the tendon healing process in humans needs further consideration, although miRNAs can generally be used as a therapeutic modality. Two miRNA-based therapeutic approaches have been devised, namely, miRNA antagonists, which mainly suppress the overexpression of pathological molecules, and miRNA mimics, which mainly reduce the expression of individual gene products but may also decrease the expression of a certain gene in in vitro experiments; thus, both approaches need to be further refined (Giordano et al. 2020).

In addition, bioengineering techniques are increasingly being used for tendon injury repair, although most of the early focus was on the three modalities of autologous, allogeneic, and xenograft transplantation, while in recent years, researchers have been conducting in-depth experiments on cell-scaffold constructs. Currently, the most commonly used bioengineering material in tendon repair is hydrogel, a biocompatible and sustainable therapeutic material that not only adheres firmly to the site of injury but also releases the corticosteroid trenbolone to reduce inflammation (Freedman et al. 2022). The connection of miRNAs with bioengineering has also been demonstrated by some researchers as a great breakthrough in miRNA application in tendon repair; for example, Yang et al. used a local continuous gene delivery system using cyclooxygenase (COX-1 and COX-2)-engineered miRNA plasmids loaded into hydrogels to greatly reduce scar adhesions in tendons, which is a major challenge in tendon repair(Yang et al. 2022). In addition, Wu et al., based on the development of tissue engineering and 3D printing technologies, loaded RNAi plasmids based on a TGF-β1 silencing microRNA (miRNA) onto 3D tendon scaffolds, a technique that not only proved to be useful for the restoration of biomechanical properties but also prevented tendon adhesion (Wu et al. 2021).

Exosome-mediated miRNA transfer is another bioengineering strategy, representing a new advancement of modern tissue engineering and regenerative medicine in tendon repair (Dinescu et al. 2021; Valadi et al. 2007). Cui et al. showed that miR-21-5p secreted by exosomes derived from bone marrow macrophages was able to target the Smad7 protein, thereby activating fibrogenesis in tendon cells and providing a rational basis for fibrosis prevention (Cui et al. 2019). In addition, Lu et al. sequenced miR-21 and found the highest expression of miR-21-3p, which is also a gene closely associated with tendon fibrosis, in human umbilical cord mesenchymal stem cells (Lu et al. 2021). Moreover, miR-29a-3p carried in umbilical cord stem cell-derived exosomes can also promote tendon healing, including promoting collagen synthesis and metabolism and cell proliferation, mainly through the mTOR/TGF-β1 signaling cascade (Yao et al. 2021). Exosome injection is a regenerative medicine approach that promotes the reprogramming of tendon cell compartments to an anti-inflammatory phenotype, thereby suppressing the inflammatory response while accelerating tendon regeneration, but there are many limitations to the use of exosomes, and experiments are needed to demonstrate whether most miRNAs can be transported to tendons using exosomes as carriers (Russo et al. 2022). In bone deficiency diseases, miRNA375 has been validated as a positive regulator promoting osteogenic differentiation, and its upregulation enhances alkaline phosphatase activity and calcium deposition. However, the application of miRNAs with bioengineering techniques has some challenges, such as the susceptibility of miRNAs to degradation by RNases, which reduces their activity, and the relatively short half-life of miRNAs, which may limit their application (Chen et al. 2019). In summary, miRNAs have been shown to play roles in angiogenesis, tendon cell differentiation, and ECM remodeling as a means to treat tendon injury. Furthermore, exosomes contain a large number of miRNAs and are the most common mode of miRNA delivery. However, the main problems in the use of miRNAs are still tissue-specific delivery and dose optimization (Tiwari et al. 2018). When these clinical problems are gradually solved, miRNAs will be a better therapeutic option for tendon repair.

## Abbreviations

miRNAs: IcroRNAs
TSPCs: Tendon stem or progenitor cells
pri-miRNA: Primary miRNA
pre-miRNA: Precursor mRNA
TRBP: TAR RNA binding protein
RISC: RNA-inducible silencing complex
Wnt: Wingless-associated integration site
mTOR: Mammalian target of rapamycin
VEGF: Vascular endothelial growth factor
ANG-1: Angiopoietin-1
PLGF: Placental growth factor
KLF6: Krüppel-like factor 6
THBS1: Thrombospondin-1
VASH1: Vasopressor-1
TGF-β: Transforming growth factor-β
α-SMA: α-Smooth muscle actin
IL-33: Interleukin-33
TNF-α: Tumor necrosis factor
HMGB1: High mobility histone-1
NCOA1: Nuclear receptor coactivator
NRIP: Nuclear receptor interaction protein
IL-6: Interleukin-6
MMP: Matrix metalloproteinase
HIF-1α: Hypoxia-inducible factorsERK1/2kinase 1/2
SCX: Helix-loop-helix transcription factor
MKX: Mohawk
EGR1: Early growth response 1
BMP: Bone morphogenetic protein
